# Response to “Co-infections in COVID-19 critically ill and antibiotic management: a prospective cohort analysis”

**DOI:** 10.1186/s13054-020-03308-4

**Published:** 2020-09-29

**Authors:** Marieke B. Nieuwenhuis, Stefaan Van Biesen, Nicole P. Juffermans

**Affiliations:** 1 Department of Intensive Care Medicine, OLVG Hospital, Amsterdam, The Netherlands; 2 Laboratory of Experimental Intensive Care and Anesthesiology, Amsterdam University Medical Center, Amsterdam, The Netherlands

To the Editor,

We read with great interest the recent paper by Verroken et al. who describe the use of rapid molecular testing in critically ill COVID-19 patients to analyse the respiratory co-infection rate [1]. We agree that rapid detection and optimized antibiotic therapy of co-infections are of great importance and commend their antibiotic stewardship. Using a multiplex PCR method, authors report an incidence of 40.6% bacterial superinfections. This incidence is not reflected in our local population and recent literature and is therefore an interesting point of discussion.

During the period of March 12 to July 27, 2020, 83 consecutive patients were admitted to the intensive care unit (ICU) of our teaching hospital in Amsterdam, the Netherlands, of whom 18 were transferred to other hospitals. Of the 65 remaining patients, 51 were intubated for mechanical ventilation. Of these, 3 died within 48 h without collection of a tracheal aspirate, leaving 48 patients of whom a tracheal aspirate was obtained. A bacterial superinfection was cultured in 6 patients (12.5%), comprising *Staphylococcus aureus* (*n* = 5) and *Pseudomonas aeruginosa* (*n* = 1). All patients received selective decontamination of the digestive tract (SDD) after admission to the ICU to reduce colonization with antibiotic-resistant gram-negative bacteria. This consisted of enteral application of amphotericin B, tobramycin and colistin during the whole ICU admission, in combination with a short initial course of intravenous cefotaxime.

Our incidence of bacterial co-infection within 48 h of ICU admission is much lower than in the reported study. A recent meta-analysis described an incidence of bacterial co-infections of 14% in ICU COVID-19 patients [2]. Explanations for the differences in microbiological infection rate could be local epidemiology of pathogens and antibiotic usage [3]. The involvement of SDD is unlikely since this is commenced after culture collection. However, an alternative explanation could be that the PCR method of Verroken et al. detected healthy microbiome of the respiratory tract, as the lungs, previously considered sterile in health, are now known to harbour microbiota.

The most relevant difference with our findings is the method of detection. We agree that adjudication of a relevant bacterial co-infection is challenging during COVID-19 infection. However, the use of PCR could potentially yield false positive results. Thereby, it is of interest which proportion of patients also had positive culture results.

## Data Availability

The datasets generated during and/or analysed during the current study are available from the corresponding author on reasonable request.

## Abbreviations

ICU: Intensive care unit
SDD: Selective decontamination of the digestive tract
